# Genome-Wide Association Studies of Root-Related Traits in *Brassica napus* L. under Low-Potassium Conditions

**DOI:** 10.3390/plants11141826

**Published:** 2022-07-12

**Authors:** Sani Ibrahim, Nazir Ahmad, Lieqiong Kuang, Ze Tian, Salisu Bello Sadau, Muhammad Shahid Iqbal, Xinfa Wang, Hanzhong Wang, Xiaoling Dun

**Affiliations:** 1 Oil Crops Research Institute of the Chinese Academy of Agricultural Sciences, Key Laboratory of Biology and Genetic Improvement of Oil Crops, Ministry of Agriculture and Rural Affairs, Wuhan 430062, China; sibrahim.bot@buk.edu.ng (S.I.); nazir_aup@yahoo.com (N.A.); kuanglieqiong@163.com (L.K.); tianze0825@163.com (Z.T.); wangxinfa@caas.cn (X.W.); wanghz@oilcrops.cn (H.W.); 2 Department of Plant Biology, Faculty of Life Sciences, College of Physical and Pharmaceutical Sciences, Bayero University, Kano, P.M.B. 3011, Kano 700006, Nigeria; 3 State Key Laboratory of Cotton Biology, Institute of Cotton Research, Chinese Academy of Agricultural Sciences (ICR, CAAS), Anyang 455000, China; sbsadau.ste@buk.edu.ng (S.B.S.); shahidkooria@gmail.com (M.S.I.)

**Keywords:** rapeseed, ML-GWAS, root-related traits, candidate gene, QTN

## Abstract

Roots are essential organs for a plant’s ability to absorb water and obtain mineral nutrients, hence they are critical to its development. Plants use root architectural alterations to improve their chances of absorbing nutrients when their supply is low. Nine root traits of a *Brassica napus* association panel were explored in hydroponic-system studies under low potassium (K) stress to unravel the genetic basis of root growth in rapeseed. The quantitative trait loci (QTL) and candidate genes for root development were discovered using a multilocus genome-wide association study (ML-GWAS). For the nine traits, a total of 453 significant associated single-nucleotide polymorphism (SNP) loci were discovered, which were then integrated into 206 QTL clusters. There were 45 pleiotropic clusters, and *qRTA04-4* and *qRTC04-7* were linked to TRL, TSA, and TRV at the same time, contributing 5.25–11.48% of the phenotypic variance explained (PVE) to the root traits. Additionally, 1360 annotated genes were discovered by examining genomic regions within 100 kb upstream and downstream of lead SNPs within the 45 loci. Thirty-five genes were identified as possibly regulating root-system development. As per protein–protein interaction analyses, homologs of three genes (*BnaC08g29120D*, *BnaA07g10150D*, and *BnaC04g45700D*) have been shown to influence root growth in earlier investigations. The QTL clusters and candidate genes identified in this work will help us better understand the genetics of root growth traits and could be employed in marker-assisted breeding for rapeseed adaptable to various conditions with low K levels.

## 1. Introduction

Potassium (K) is an imperative element for plants that is crucial for crop growth, development, reproduction, and crop production and quality [1]. It helps plants improve their tolerance to salt, drought, hot and cold stress, disease, etc. [2,3,4,5,6]. Under water stress, high K application has been demonstrated to improve the photosynthetic rate, plant development, and yields of numerous crops [7]. In addition, during drought stress, inadequate potassium causes a reduction in photosynthesis and biomass, and maintaining high K in tissues is linked to drought tolerance [8,9]. Although potassium (K) reserves account for 2.6% of the earth’s crust, only 0.1–0.2% K inside the soluble form is bioavailable to plants [10]. Breeding high-K-efficiency crops is of great significance for improving soil K utilization rate, increasing crop yield, and reducing K fertilizer application.

The root system is where nutrients and water are acquired, and is composed of root length, root branches, root angle, root diameter, root mass, root hair, and other specific root characteristics [1,11,12]. Nevertheless, according to extensive studies, root morphological (RM) features such as root length, surface area, volume, and quantity have a substantial impact on K uptake and are flexible and responsive to K availability [13,14]. Undoubtedly, enhancing plant root architecture to increase root K absorption activity could be a way of improvement of plant K utilization efficiency (KUE) [15]. Root research and breeding activities that aim to improve root qualities have been identified as key aims for ensuring the high yields required to feed an ever-increasing population of humans [16,17]. As a result, deciphering the genetic basis of RM characteristics and increasing root K absorption activity in the LK condition will considerably improve KUE and improve the long-term productivity of crops [15].

Recently, genome-wide association studies (GWAS) have emerged as a promising alternative to QTL mapping because they capture more variety and provide better resolution for gene and favorable allele findings [18]. Theoretically, GWAS evaluates the extent of the link between a genotype and a phenotype using statistics and then suggests genes and alleles that are associated with specific features [19]. More subsequently, GWAS has shown effectiveness in several crop varieties, including peanut [20], wheat [21], oat [22], cotton [23], rice [24], guinea corn [25], Maize [26], soybean [27], and barley [28]. Single-locus models such as the general linear model (GLM) and the mixed linear model (MLM) were used in the GWAS [29]. Because multilocus models do not need the Bonferroni adjustment, they are the best alternative for GWAS because they can identify more marker–trait associations [23]. Several multilocus GWAS models have recently been introduced, including multilocus RMLM (mrMLM) [30], fast multilocus random SNP-effect EMMA (FASTmrEMMA) [31], and iterative modified-sure independence screening EM-Bayesian LASSO (ISIS EM-BLASSO) [32].

Rapeseed (*Brassica napus* L.; AACC, 2*n* = 38), behind soybean and palm oil, is the world’s third-largest oil crop. It was evolved from natural interspecies hybridization between two phylogenetically distant species, *B. rapa* (AA, 2*n* = 20) and *B. oleracea*, about 10,000 years ago [33,34]. Rapeseed absorbs between 290 to 373 kg ha^−1^ of K, with the majority of the K coming from the soil. Some studies in *B. napus* have focused on root traits, specifically the root development at the seedling stage; root vigor; root development at the mature stage; root growth dynamics; variations in root growth habits of spring, winter, and semi-winter rapeseed; and the significance of root morphology in terms of phosphorus and nitrogen absorption [35,36,37,38,39,40,41,42,43]. So far, few studies have identified genetic pathways or genes associated with the architecture of the root system in *B*. *napus* grown in low-potassium environments.

In the present study, we used six multilocus models for the GWAS of root-related traits in *B. napus* in hydroponic-system trials with low K treatments, based on a 50 K *Brassica* Infinium SNP array used to genotype an association panel of 327 *B. napus* accessions, including spring, winter, and semi-winter. The findings revealed the entire genome of QTL for marker-assisted selection in breeding strategies and the genetic basis of root-related traits in rapeseed.

## 2. Results

### 2.1. Performances of Eight Lines under K-Concentration Gradients

Eight lines, H1, H2, H3, H4, H5, H6, H7, and H8, randomly selected from the *B. napus* association panel were grown hydroponically and analyzed in two independent trials under nine K concentrations, from K1 to K9 with 6, 0.6, 0.3, 0.15, 0.075, 0.05, 0.025, 0.01, and 0.005 mM K^+^, respectively. The shoot fresh weight (SFW) of the eight lines were investigated to evaluate the effects of different potassium deficiency on rapeseed growth (Figure S1). As shown in Figure S1A,B, the SFW of almost all materials was decreased with the decrease in treatment concentrations, showing that the K stress has a great influence on plant growth. At the 0.01 mM K^+^ (K8), when contrasted to the control condition (6 mM K^+^), the SFW of all the eight lines had a significant decline, with the ratio of SFW less than 50% (Figure S1B). Furthermore, the ratio of SFW of the eight genotypes displayed significant differences, with the values ranging from 15.5% to 46.0% (Figure S1B), implying considerable variations in potassium-uptake efficiency among *B. napus* genotypes.

### 2.2. Phenotypic Variations of Root Traits in the Association Panel under Low-K Stress

According to the results of K-concentration-gradient treatments, the 327 natural accessions in the *B. napus* association panel were studied under low-K stress (the concentration of K was 0.01 mmol L^−1^) by hydroponics to determine the genetic mechanism underlying rapeseed variations in root-related traits under the low-K treatment. The nine root-related traits and shoot biomass traits, PRL, SFW, RFW, TRL, TSA, TRV, TRN, TFW, and RSR, were evaluated (Table 1).

**Figure 1:**
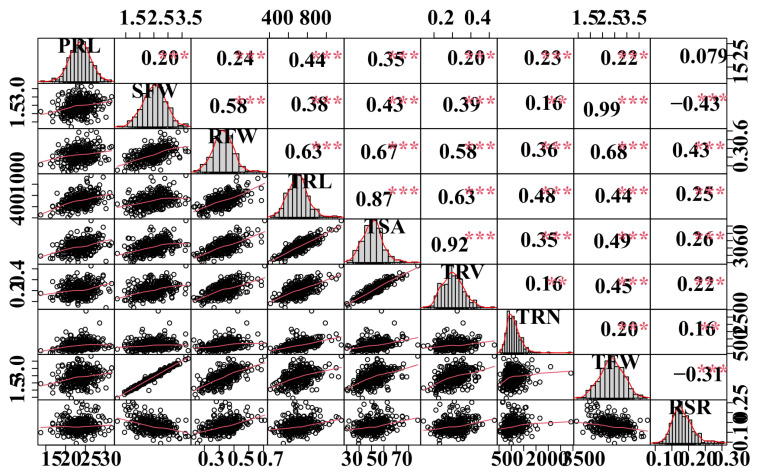
**Correlation analyses between root-related traits.** The plots on the diagonal line show the frequency distribution of the traits. Above the diagonal line are Pearson correlation coefficient values between traits, and the plots below the diagonal line indicate the scatter plots of the root-related traits. **, *** are significant differences at *p* < 0.05, *p* < 0.01, and *p* < 0.001, respectively. Refer to Table 1 for the definition of the terms.

The coefficient of variation (CV) values for all traits in the population ranged from 9.95% to 61.34%, indicating significantly phenotypic variations. The broad-sense heritability (H^2^) of all measured traits varied between 49.4% to 60.4%. PRL, RFW, TRN, and TRL exhibited approximately higher heritabilities than TRV (Table 2), implying that genetic factors account for most of the phenotypic variability for these variables in *B. napus*. Phenotypic correlation analysis revealed that most measured traits were strongly associated, except for PRL (Figure 1). SFW, RFW, TRL, and TSA all had strong positive correlations (r = 0.58–0.99, *p* < 0.001) (Figure 1).

Principal-component analyses were performed for the nine traits, and two major principal components were discovered, accounting for more than 60% of the phenotypic variance (Figure S2). TRN, PRL, RFW, SFW, TRL, TSA, and TRV were among the seven traits for which the first principal component (PC1) accounted for 48.64% of the variability. PC2 accounted for 20.67% of the variation and was primarily responsible for RSR (Figure S2). The findings supported the phenotypic correlation analysis. Furthermore, all of the traits varied significantly across the 327 genotypes, and they differed considerably under the genotype–environment interaction (G × E) (*p* < 0.001; Table S1) according to the analysis of variance (ANOVA).

### 2.3. QTL Clusters Related to the Root System under Low-K Stress Were Obtained by GWAS

**Figure 2:**
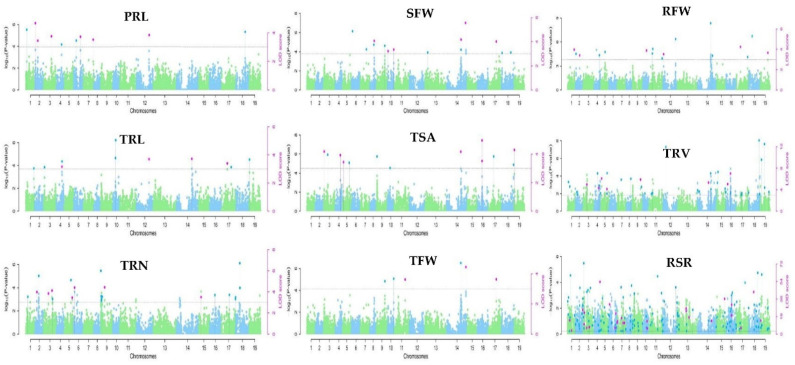
Manhattan of phenotype–genotype association analysis using six multilocus GWAS approaches for nine root-related traits of *B. napus*. The 19 chromosomes from A01–A10 and C01–C09 are represented by varied green and blue colors in the plots; the thick horizontal lines represent the logarithm of odds (LOD) threshold of ≥3. Significant SNPs are indicated by colored dots above the threshold values. The green and blue dots above the threshold line represent significant SNPs by the threshold (log_10_1/21,242 = 4.33 × 10^−5^, while the pink dots show significant SNPs by the threshold LOD ≥ 3.

This study identified 453 significant marker–trait associations (MTAs) for root-related traits under low-K stress using six multilocus GWAS methods (mrMLM, FASTmrMLM, FASTmrEMMA, pLARmEB, pKWmEB, and ISIS EM-BLASSO) in the mrMLM package, based on a logarithm of odds (LOD) threshold of ≥3 (Table S2). Manhattan plots (Figure 2) and QQ plots (Figure S2) were used to depict the significant MTAs related to each trait. On chromosome C07 (chromosome 17), common significant SNPs for the examined traits SFW, RFW, TRL, TFW, and RSR were discovered (Figure 2). This suggested that several root-related traits were controlled by genetic variants at this locus.

Furthermore, if the peak SNPs with near vicinity (within 1 Mb) and have an LD r^2^ > 0.2, they are categorized as the same QTL [38]; therefore, the 453 significant MTAs were integrated into 206 QTL clusters (Table S3). These QTL clusters were distributed on all the 19 *B. napus* chromosomes with the highest number on C04 and C06. In addition, 45 pleiotropic loci contributed 12.56% of the phenotypic variance explained (PVE) to the root traits (Table S4). Two QTN clusters, *qRT.A04-4* and *qRT.C04-7*, were observed to be associated with four traits. Intriguingly, the two QTL clusters both influenced three similar traits; TRL, TSA, and TRV, and contributed PVE of 5.23% to 11.48% to the traits. These key pleiotropic loci discovered in this study could improve nutrient uptake and utilization efficiency, and these traits could be exploited to enhance rapeseed root-system architecture.

Moreover, *qRT.A01-8*, *qRT.A02-11*, *qRT.A06-10*, and *qRT.C06-5* influenced similar traits, TRN and RSR, and contributed PVE of 1.6% to 5.07% to the traits. These traits are critical for RSA as they enable the root to attach and partake in the absorption of nutrients by increasing the volume of soil covered by the root. The clusters *qRT.A03-12*, *qRT.A09-14*, *qRT.C01-7*, and *qRT.C07-6* related to TSA, TRV, TRN, TRL, RFW, and RSR explained 1.32–5.03% of the phenotypic variance. This finding suggested that targeting these traits might be particularly beneficial in enhancing K uptake in breeding programs.

Furthermore, TRL, TSA, and TRV of germplasm with favorable alleles (AA; *n* = 194, 18, 192, and 194) were 59.33%, 57.80%, and 58.72%, respectively, at marker Bn-A04-p12314909, which were greater than 38.23%, 36.39%, and 37.31% of genotypes with unfavorable alleles (GG; *n* = 125, 119, and 122). Furthermore, at marker Bn-scaff 16394 1-p850210, the proportion TRL, TSA, and TRV of genotypes with favorable alleles (AA; *n* = 246, 249, and 245) were 75.23%, 76.15%, and 75.84%, respectively, compared to 9.48%, 9.17%, and 8.87% for genotypes with unfavorable alleles (CC; *n* = 31, 30, and 29).

### 2.4. Candidate Genes Associated with Root-Related Traits

To identify candidate genes relevant to root development, we extracted all the genes within the 100 kb window (LD area) around every peak SNP within the main 45 QTL clusters. A total of 1360 annotated gene models have been detected in the *B. napus* ‘*Darmor*’ genome, as per gene annotation (Table S5). The number of genes found near the peak SNPs varied from 12 to 52. Based on the annotation information of the retrieved genes and the functions defined for their homologs in *Arabidopsis thaliana*, the function of the corresponding gene was inferred. *AtP5CS2* is encoded by *BnaA09g35230D*, which was discovered 3.722 kb downstream of the peak SNP seq-new-rs26492 of *qRT.A09-4* and is one of the 35 candidate genes (Table 3). Two Arabidopsis paralog genes, *P5CS1* and *P5CS2*, encode the first enzyme in this pathway, which catalyzes the rate-limiting step of proline synthesis in higher plants [44]. In the early phases of postembryonic root growth, proline appears to govern cell proliferation [45]. The Arabidopsis homolog of the *PIN-FORMED 7* (*PIN7*) gene, which is located 25.949 kb upstream of the lead SNP seq-new-rs32620 of *qRT.A07-6*, is *BnaA07g10150D* (Table 3). *PIN* auxin efflux carriers have a variety of roles in root-system radial extension according to the developmental framework [46]. *BnaC08g29120D* was identified 41.239 kb upstream of the *qRT.C08-5* marker seq-new-rs34390, a homolog of Arabidopsis *Lateral organ boundaries-domain 29* (*LBD29*). According to Porco et al. [47], disrupting auxin-inducible *LBD29* expression or generating an *LBD29-SRDX* transcriptional repressor phenocopied the *lax3* mutant and resulted in slowed lateral root appearance. *BnaA09g03750D* is also a homolog of Arabidopsis *AUXIN RESISTANT 1 (AUX1)*, and it was discovered 33.579 kb away from the *qRT.C04-2* peak marker Bn-scaff_16888_1-p1169101 (Table 3), and *AUX1* is implicated in the modulation of ethylene during root gravitropism, according to Fei et al. [48]. *AUX1* appears to be essential for maintaining temperature-regulated root gravitropism in *ckrc1-1*.

### 2.5. GO and KEGG Analysis of Potential Candidate Genes

On the 35 candidate genes, we performed GO enrichment analysis and KEGG pathways analysis to understand more about their functions (Table S6). In the three GO categories of biological process, cellular component, and molecular function, they were well-represented. The most regularly utilized GO terms at (*p* < 0.05) are shown in Figure S4A,B. The auxin-activated signaling pathway and primary root development were the most abundant GO terms in the biological process category. The most abundant GO terms in the cellular component category were SCF ubiquitin ligase and the cytosolic proteasome. Auxin binding and cytokinin dehydrogenase activity were the most abundant GO terms in the molecular function category.

*BnaC08g17490D*, the Arabidopsis homolog of *UDP-glucose pyrophosphorylase 2* (*UGP2*), is implicated in galactose metabolism, according to the KEGG pathway study (Figure S4C). In the presence of D-galactose, the root-shortening and epidermal-bulging phenotypes in *rhd1* were completely suppressed, whereas the WT root morphology was fully restored [49]. Also implicated in arginine and proline metabolism is the Arabidopsis homolog of *delta 1-pyrroline-5-carboxylate synthase 2*, *BnaA09g35230D*. Proline can influence the size of the root meristematic zone in Arabidopsis, according to Biancucci et al. [45], possibly influencing cell division, and as a result, the ratio between cell division and cell differentiation.

### 2.6. Protein Interaction Network Analysis, Phylogenetic Trees, Gene Structure Analysis, and Motif Analysis

We utilized STRING (http://string-db.org/cgi/ (accessed on 15 October 2020)) to develop a protein interaction network that included all 1360 genes in the LD region around each lead SNP within the 45 pleiotropic QTN clusters to further explore the gene’s functional connections. There were 62 nodes and 34 edges in the network. The GWAS candidate genes were represented by purple, green, red, and blue nodes in the network (Figure S5). *BnaC08g29120D*, *BnaA09g35230D*, *BnaA03g06830D*, *BnaC04g45700D*, and *BnaA07g10150D* all revealed significant interactions or might play crucial roles in the interaction through interacting with other genes.

In the interaction networks, 31 candidate genes were connected, according to the protein–protein interaction analysis (Figure S5). *BnaC08g29120D*, *BnaA09g35230D*, *BnaA03g06830D*, *BnaC04g45700D*, and *BnaA07g10150D* were revealed to have significant connections and may play essential roles in the same family via interacting with other genes. The gene *BnaC08g29120D*, specifically, could be important in the networks. We noticed that the gene *BnaC08g29120D* was critical in the networks, in addition to the huge linkage. *LBD29*, the Arabidopsis homolog of *BnaC08g29120D*, is a crucial node in the lateral root-emergence control network downstream of auxin and *ARF7*, as previously described [47]. The subsequent polarization of *PIN7*, which is encoded by *BnaA07g10150D*, signifies the bending towards gravity, and hence the escape from the plateau phase. This developmental approach elucidates the many roles of PIN auxin efflux carriers in regulating root-system radial growth [46]. According to Fei et al. [48], *AUX1*, the Arabidopsis homolog gene of *BnaC04g45700D* is involved in the interaction between auxin and ethylene, as well as interaction-mediated polar auxin transport, during plant root development in response to increased ambient temperature. This result shows that the key genes should be further explored to learn more about their potential roles in the network.

**Figure 3:**
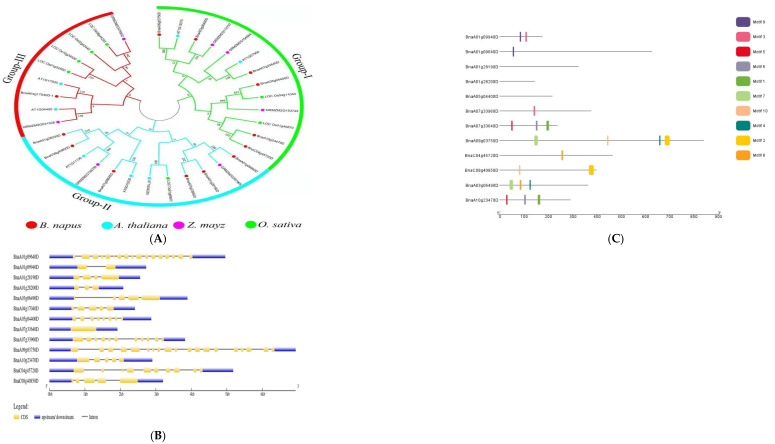
Analysis of candidate genes. (**A**) Genes from *B. napus*, *A. thaliana*, *Oryza sativa*, and *Zea mays* are shown in a phylogenetic tree with subgroups. The phylogenetic tree was built with MEGA 7 software using the neighbor-joining method with a bootstrap value of 1000. The bootstrap values are indicated by the numbers next to branches. (**B**) The distribution of exons and introns in the genes. (**C**) Motif analysis of candidate genes in *B. napus*. The various colors represent the various sorts of motifs that the genes possess.

Phylogenetic analysis of genes from *B. napus*, *A. thaliana*, *Zea mays*, and *Oryza sativa* was used to classify the potential candidate genes. The genes were divided into three groups using the terms Groups I, II, and III. The largest group, Group I, had 13 individuals, followed by Groups II and III, which had twelve and nine members, respectively. Six of the *B. napus* genes were detected in each of group I and II, but only one gene was found in Group III (Figure 3A). Li et al. [38] and Ibrahim et al. [37] classified genes from rapeseed and three other plant species, including *Brassica rapa*, *Brassica oleracea*, and *Arabidopsis thaliana*, into four main groups. To learn more about gene-structure evolution and structural features, the putative genes’ coding sequences were aligned to the genomic sequences. According to the findings, the majority of the genes had several exons and introns; however, two genes, one without an intron and the other with only one intron, had only one intron in their coding regions. The protein lengths of all of the genes were different, indicating that the discrepancies in their gene structures are not only due to changes in intron numbers and lengths (Figure 3B). It is usually thought that stress-response genes have a small number of introns [50]. Consequently, stress reactions are virtually certainly linked to these genes.

Researchers looked at motif patterns to understand more about the structural evolution of *B. napus* proteins. Using the MEME tool, a total of ten distinct motifs were found. The identified motifs revealed that motif 9 was conserved in both Bna09940 and Bna09640; however, motif 3 was conserved in both Bna09940 and Bna33960 but not in 09640. Motifs 10 and 2 were conserved in Bna03750 and Bna40850, respectively; however, motifs 7 and 4 were not conserved in Bna4085. Furthermore, in Bna33640 and Bna23470, motifs 5, 6, and 1 were conserved, whereas motif 8 was conserved in Bna45720 and Bna06490, but motifs 7 and 4 were not. None of the ten motifs were found in Bna28190, Bna28200, or Bna04400 (Figure 3C). This suggests that these genes may be involved in the complementing roles linked with a variety of abiotic inputs. Li et al. [51] have also described similar discoveries. Phylogenetic investigations revealed that several genes in some groups had similar patterns of conserved motifs, hinting that these conserved motifs are significant in the group/groups-specific functions.

## 3. Discussion

Root development is a crucial trait that affects crop output and quality. Mapping the genetic loci for root growth traits could aid in revealing the genetic basis for root growth and may be useful in the development of cultivars that are adaptable to various geographical locations [52]. In rapeseed, Arabidopsis, rice, wheat, barley, and maize, GWAS has successfully identified thousands of root-associated loci, as well as multiple significant genes and variations [53]. Using the six multilocus approaches in the mrMLM package, this study found 453 significant MTAs for nine root traits using 21,242 SNPs identified in the association panel. Natural variations in complex traits were successfully detected using the GWAS approach. The variation could be explained by differences in evolutionary time and mating systems. Intrachromosomal LD is impaired by recombination, whereas mutation contributes the raw material for producing LD variations [54]. The heritability of the traits in this study was modest, ranging from 49.37% to 62.09%, implying that these traits were equally important in plant K uptake.

Our genotype collection included 327 rapeseed genotypes from more than four countries on three continents, demonstrating a sample population with sufficient genetic variation for a GWAS. Population structure (Q) and kinship are common causes of false-positive outcomes in association analysis (K). Corrected MLM, which considered both the Q and K matrices, performed better than either the Q or the K alone [55]. However, in several cases, the MLM (Q + K) model was too stringent, and important SNPs were ignored. To minimize the occurrence of false positives, we used six multilocus techniques from the mrMLM package. Multilocus GWAS models are better than single-locus GWAS models, which seem to be closer to actual genetic models of plants and animals, due to their higher statistical power and lower FPR [30].

Hundreds of root QTL have been identified in controlled environments or the field in several investigations [15,41,43,56,57,58]. Moreover, many of these QTLs have been linked to traits such as yield, water/nutrient absorption, and abiotic stress tolerance [57,59,60,61,62]. In this work, the peak SNPs of 15 QTN clusters were found in the same haplotype blocks as the 27 previously reported important SNPs associated with *B. napus* root-development dynamics (Table S8; [38]). Nonetheless, previously identified QTL (*qcA02-1*, *qcA02-2*, and *qcA02-3*) associated with total root number, shoot dry weight, and root–shoot ratio in dry weight discovered under low-nitrogen conditions were likewise colocalized with our cluster *qRT.A02-4* [15,41]. Under high-nitrogen conditions, the clusters *qRT.A04-4* and *qRT.A04-5* were also colocalized with qcA04, which is linked to shoot fresh and dry weight [15]. Our findings revealed beneficial QTNs that can be utilized to select excellent root traits in rapeseed using marker-assisted selection.

Favorable alleles were described as SNP alleles with more of these alleles that enhance root development, whereas “unfavorable alleles” were described as SNP alleles with fewer of these alleles that enhance root development, including heterozygous alleles [63]. Favorable and unfavorable alleles can be found in certain species with simple genetic backgrounds without taking heterozygous SNPs into account [64]. Our result is consistent with the findings of a similar study in *B. napus* [37], which found that the proportion of TRL, TSA, and TRV of germplasm with favorable alleles was higher than the proportion of TRL, TSA, and TRV of germplasm with unfavorable alleles. These results revealed that the genetic modulation of root development in *B. napus* has a largely multiplicative effect.

## 4. Materials and Methods

### 4.1. Plant Materials

The association panel used in this study was provided by Oil Crops Research Institute, CAAS, Wuhan, China, comprising 327 *B. napus* genotypes collected from breeding institutes based on the Rapeseed Research Network in China, comprising winter, semi-winter, and spring accessions. According to the growing habits of accessions, they usually grow in China throughout the winter. Eight *B. napus* genotypes from the association panel, consisting of H1, H6, H7, and H8 (semi-winter accessions); H2 and H3 (winter accessions); and H4 and H5 (spring accessions), were used for the pilot experiment to find the suitable concentration of potassium, the results of which were used for low-potassium treatment of the population.

### 4.2. Experimental Design and Growth Condition

The eight lines were grown hydroponically and analyzed in two independent trials with a completely random design at the Chinese Academy of Agricultural Sciences’ Oil Crops Research Institute in Wuhan, China. Hogland’s solution [65], with consistent concentrations of other elements, and nine potassium concentrations were used, including 6, 0.6, 0.3, 0.15, 0.075, 0.05, 0.025, 0.01, and 0.005 mM K^+^. The full-strength modified Hoagland’s solution contained 5 mmol L^−1^ Ca (NO_3_)_2_.4H_2_O, 5 mmol L^−1^ KNO_3_, 2 mmol L^−1^ MgSO_4_.7H_2_O, 1 mmol L^−1^ KH_2_PO_4_, 0.05 µM EDTA-Fe, 46 µM H_3_BO_3_, 14 µM MnCl_2_.4H_2_O, 0.77 µM ZnSO_4_.7H_2_O, 0.32 µM CuSO_4_ and 0.44 µM Na_2_ MoO_4_.2H_2_O. Quarter-strength, half-strength, and full-strength nutritional solutions were applied for the first, second, and third week, respectively. The seeds were planted on the germination device’s medical gauze, maintained in the dark for two days, and then exposed to light for four days in a greenhouse at 26 °C for 16 h, 180 µmol photons m^−2^s^−1^ daylight light intensity, and 21 °C for 8 h, 50–70% relative humidity. The seedlings were then selected and transferred into small blue plastic basins (34 cm in length, 26 cm wide, and 12 cm in height). Twelve seedlings were grown for each genotype, and the nutrient solution was challenged regularly.

The 327 association panels were grown hydroponically and analyzed in three independent trials with a completely random design at the Chinese Academy of Agricultural Sciences’ Oil Crops Research Institute in Wuhan, China. With consistent concentrations of other elements, two concentrations of control treatments (control, 6 mmol L^−1^) and stress (stress, 0.01 mmol L^−1^) were applied. Six seedlings were grown for each genotype, and the nutrient solution was changed regularly.

### 4.3. Root Phenotyping

Three plants from each genotype were collected 21 days after transplantation, and each plant was dissected into the root and shoot sections. The primary root length (PRL), shoot fresh weight (SFW), and root fresh (RFW) weight were manually determined, and the entire root system was placed in a water-filled plastic tray and scanned with (EPSON V700, Nagano, Japan). Using WinRHIZO software (Pro 2012b, Quebec City, QC, Canada), the total root length (TRL), total root volume (TRV), total root number (TRN), and total root surface area (TSA) of the roots were analyzed. Total fresh weight (TFW) was determined as the total of fresh weights (SFW + RFW), and root–shoot ratio (RSR) was determined as the ratio of fresh weights (RFW/SFW).

### 4.4. Data Analysis

The data were analyzed using analysis of variance (ANOVA) based on the generalized linear model, and the mean values of each genotype were employed for statistical analysis (GLM). The “PerformanceAnalytics” package in R software was used to calculate Pearson correlation at a significance level of (*p* < 0.05). For each variable, the broad-sense heritability (H^2^) was estimated [66] as follows: H^2^ = (σ2G)/ (σ2P), σ^2^G = (MSG − MSE/rep), σ^2^P = (MSG − MSE/rep) + MSE; H^2^ = (MSG − MSE/rep)/(MSG − MSE/rep) + MSE. The genotypic and phenotypic variations are represented by σ^2^G and σ^2^P, respectively. MSG and MSE are the mean square of genotype and mean-square error, respectively, computed by analysis of variances (ANOVA) using the software SAS 9.2 (SAS Institute Inc., Cary, NC, USA), and rep is the number of replications.

### 4.5. Marker–Trait Association

The 327 *B*. *napus* germplasms were genotyped by a *B. napus* 50 K Illumina Infinium [37]. The 21,242 SNP markers developed for the 327 *B*. *napus* germplasms were used in a genome-wide association analysis for root-system architectural traits. Six multilocus GWAS models (mrMLM, FASTmrMLM, FASTmrEMMA, pLARmEB, pKWmEB, and ISIS EM-BLASSO) were used in the R mrMLM (v4.0.2) package [30]. The kinship matrix (K matrix) was generated utilizing genetic marker information. A logarithm of odds (LOD) threshold of ≥3 was used to identify statistically significant loci [23].

### 4.6. Exploration of Candidate Genes

The haplotype blocks were investigated by haploview 4.2 [67], and the LD blocks were defined by the four-gamete rule with a fourth haplotype-frequency cutoff of 0.001 to define regions of interest for the identification of candidate genes. To identify candidates, all genes within the LD block were considered. The 100-kb flanking regions on either side of significantly associated markers within the LD blocks were used to identify the candidates.

### 4.7. GO and KEGG Analysis

The 1360 possible candidate genes were analyzed for gene ontology (GO) enrichment and Kyoto Encyclopedia of Genes and Genomes (KEGG) pathway enrichment using the Oebiotech website (https://cloud.oebiotech.cn/task/ (accessed on 13 February 2022)), and the significantly enriched GO terms were found at (*p* < 0.05).

### 4.8. Protein Interaction Network Analysis, Phylogenetic Trees, Gene-Structure Analysis, and Motif Analysis

To further examine the gene’s functional interactions, we then used the Internet application STRING (http://string-db.org/cgi/, accessed on 11 September 2021) to establish a protein interaction network with all the GWAS genes retrieved from the LD region around each peak SNP. GSDS (http://gsds.cbi.pku.edu.cn, accessed on 11 September 2021) was utilized to perform the gene-structure analysis. The protein sequences of numerous plant homologous genes were acquired from the appropriate plant databases. ClustalX software version 1.2, EMBL, Heidelberg, Germany was used to examine sequence alignment [68]. With MEGA 7.0 software, an NJ phylogenetic tree was created using the bootstrap procedure with 1000 replications based on protein sequences. Using the online program WolfPSORT (https://wolfpsort.hgc.jp, accessed on 8 September 2021) for predicting subcellular protein localization, protein sequences of the candidate genes were used to estimate subcellular localization.

## 5. Conclusions

In this regard, we used six multilocus GWAS model analyses to genetically unravel root-related traits in hydroponic-system experiments under low-K treatments to uncover genetic regions and candidate genes influencing root growth traits in *B. napus*. In total, 453 significant MTAs were discovered for root-related traits. The A03 and C04 chromosomes were shown to have the most influence on root growth. We uncovered 45 crucial pleiotropic loci linked to root traits in rapeseed that might be used to enhance RSA. Thus, 17 of these were discovered to overlap with reported earlier QTLs, hence they were chosen for further research. Moreover, within a 100 kb region of these substantial marker–trait associations of the 45 important loci, 35 orthologs of functional candidate genes linked to root growth were discovered. *BnaA04g16140D*, *BnaA04g16400D*, and *BnaA05g04380D* were shown to be linked to TRL, TSA, TRV, and RSR suggesting that *BnaA04g16140D*, *BnaA04g16400D*, and *BnaA05g04380D* may be the most important candidate effect gene for rapeseed root development in this study. These substantial QTN clusters and candidate genes could provide potential avenues for rapeseed root growth and development of molecular breeding and functional research. Our results are important resources for future research into the molecular pathways implicated in rapeseed root development and crop improvement under low-K conditions. Because finding causal genes is challenging, additional research is needed to explore the molecular functions of these candidate genes through more extensive studies.

## Figures and Tables

**Table 1:** Information on the 9 evaluated traits.

Classification	Trait Description	Abbreviations	Units
Root-related traits	Primary root length	PRL	cm
	Total root volume	TRV	cm^3^
	Total root surface area	TSA	cm^2^
	Total root length	TRL	cm
	Total root number	TRN	N
Biomass-related traits	Root fresh weight	RFW	g
	Shoot fresh weight	SFW	g
	Total fresh weights	TFW	g
	Root–shoot fresh weight ratio	RSR	

**Table 2:** Statistics for 327 *B. napus* genotypes with 9 traits examined.

Trait	Mean	SD	Min	Max	Skewness	Kurtosis	CV (%)	H^2^ (%)
PRL	23.57	2.77	13.72	31.62	−0.05	0.35	11.73	56.9
SFW	2.47	0.44	1.20	3.72	0.01	−0.07	17.83	60.4
RFW	0.43	0.08	0.23	0.71	0.28	0.65	18.38	60.3
TRL	711.5	115.1	409.8	1156.3	0.47	1.16	16.17	53.1
TSA	51.7	8.54	30.2	87.4	0.42	0.94	16.51	52.6
TRV	0.31	0.06	0.14	0.53	0.29	0.17	19.15	49.4
TRN	1084.1	310.5	540.3	3417.2	2.22	11.39	28.64	52.1
TFW	2.90	0.49	1.43	4.35	−0.01	−0.12	17.03	60.2
RSR	0.18	0.03	0.09	0.31	0.89	1.54	18.27	62.1

**Table 3:** Candidate genes for different root traits within 100 kb region at either side of the lead SNP.

Cluster	Trait	Lead SNP	Position (bp)	Gene Model	Distance to Lead SNP (Kb)	Gene Symbol	At Homolog Genes	Annotation
*qRT.A02-4*	RFW, RSR	Bn-A02-p13803435	10,432,009	BnaA02g17300D	−29.99	*SAUR*	AT1G75580	SAUR-like auxin-responsive protein family
*qRT.A02-11*	TRN, RSR	Bn-A02-p8199891	5,191,339	BnaA02g10340D	−94.04	*CRF3*	AT5G53290	Cytokinin response factor 3
*qRT.A03-12*	TSA, TRV, RSR	Bn-A03-p10023639	9,214,456	BnaA03g19500D	−22.93	*CKX1*	AT2G41510	Cytokinin oxidase/dehydrogenase 1
*qRT.A03-17*	TRL, TSA	seq-new-rs32620	2,981,029	BnaA03g06500D	76.26	*UGP2*	AT5G17310	UDP-glucose pyrophosphorylase 2
				BnaA03g06730D	−35.11	*MYB56*	AT5G17800	MYB domain protein 56
				BnaA03g06800D	−61.10	*SAUR*	AT5G18010	SAUR-like auxin-responsive protein family
*qRT.A04-4*	RSR, TRL, TSA, TRV	Bn-A04-p12314909	13,306,243	BnaA04g16140D	97.43	*GH3*	AT1G48670	Auxin-responsive GH3 family protein
*qRT.A05-11*	TSA, TRV	Bn-A05-p2142102	2,273,504	BnaA05g04380D	−85.25	*ERF13*	AT2G44840	Ethylene-responsive element binding factor 13
*qRT.A06-10*	RSR, TRN	seq-new-rs33430	2,265,805	BnaA06g03580D	71.58	*GH3*	AT1G23160	Auxin-responsive GH3 family protein
				BnaA06g03620D	33.41	*RACK1B_AT*	AT1G48630	Receptor for activated C kinase 1B
*qRT.A07-1*	PRL, RSR	Bn-A07-p21573107	23,110,821	BnaA07g33740D	25.48	*ARF17*	AT1G77850	Auxin response factor 17
*qRT.A07-6*	TRV, RSR	seq-new-rs40608	9,704,794	BnaA07g10150D	25.95	*PIN7*	AT1G23080	PIN-FORMED 7
*qRT.A08-1*	RSR, SFW	seq-new-rs39127	12,905,937	BnaA08g15590D	−35.17	*CP1*	AT4G36880	Cysteine proteinase1
				BnaA08g15600D	−56.19	*GASA1*	AT1G75750	GAST1 protein homolog 1
*qRT.A09-4*	TFW, SFW	seq-new-rs26492	25,679,360	BnaA09g35190D	21.94	*SDIR1*	AT3G55530	SALT- AND DROUGHT-INDUCED RING FINGER1
				BnaA09g35230D	−3.72	*P5CS2*	AT3G55610	Delta 1-pyrroline-5-carboxylate synthase 2
*qRT.A10-1*	SFW, TFW	Bn-A10-p15967013	15,593,735	BnaA10g23640D	16.74	*GA20OX3*	AT5G07200	Gibberellin 20-oxidase 3
				BnaA10g23650D	9.42	*RR21*	AT5G07210	Response regulator 21
				BnaA10g23740D	−42.10	*ML4*	AT5G07290	MEI2-like 4
*qRT.A10-3*	RSR, RFW	Bn-A10-p10120142	11,533,850	BnaA10g14470D	28.88	*EIL2*	AT5G21120	ETHYLENE-INSENSITIVE3-like 2
*qRT.C01-7*	RSR, TRV, RFW	seq-new-rs38417	12,806,484	BnaC01g18450D	−24.27	*LTI65*	AT5G52300	LOW-TEMPERATURE-INDUCED 65
*qRT.C04-2*	SFW, TFW	Bn-scaff_16888_1-p1169101	45,355,403	BnaC04g45700D	33.58	*AUX1*	AT2G38120	AUXIN RESISTANT 1
				BnaC04g45720D	23.19	*CAX1*	AT2G38170	Cation exchanger 1
				BnaC04g45770D	−11.70		AT2G38240	2-oxoglutarate (2OG) and Fe(II)-dependent oxygenase superfamily protein
*qRT.C04-8*	RSR, TRV	seq-new-rs30573	25,264,080	BnaC04g24310D	−42.70	*EIF3E*	AT3G57290	Eukaryotic translation initiation factor 3E
*qRT.C06-10*	TRV, RSR	seq-new-rs48045	5,209,723	BnaC06g04590D	−14.14		AT1G51460	ABC-2 type transporter family protein
				BnaC06g04620D	−63.33		AT1G51538	Aminotransferase-like, plant mobile domain family protein
*qRT.C07-1*	TRV, TRN	seq-new-rs28637	44,197,394	BnaC07g46640D	28.74	*LCR59*	AT4G30070	Low-molecular-weight cysteine-rich 59
*qRT.C07-2*	RSR, TRL	seq-new-rs46639	35,175,730	BnaC07g30830D	55.17	*LR*	AT5G23400	Leucine-rich repeat (LRR) family protein
				BnaC07g30930D	−19.94	*SAR1*	AT1G33410	SUPPRESSOR OF AUXIN RESISTANCE1
*qRT.C07-6*	TRL, TSA, TRV	Bn-scaff_18202_1-p1536412	22,287,865	BnaC07g16350D	−20.37	*ABA1*	AT5G67030	ABA DEFICIENT 1
*qRT.C08-5*	RSR, SFW	seq-new-rs34390	29,522,206	BnaC08g29060D	64.89	*AFB3*	AT1G12820	Auxin signaling F-box 3
				BnaC08g29120D	41.24	*LBD29*	AT3G58190	Lateral organ boundaries-domain 29
*qRT.C09-1*	TRV, RSR	seq-new-rs41567	42,567,030	BnaC09g40100D	−55.68	*WOX12*	AT5G17810	WUSCHEL related homeobox 12
*qRT.C09-2*	RFW, RSR	seq-new-rs25004	38,586,454	BnaC09g35190D	−14.05		AT5G59845	Gibberellin-regulated family protein

## Data Availability

The datasets generated during and/or analyzed during the current study are available from the corresponding author on reasonable request.

## Supplementary Materials

The following are available online at https://www.mdpi.com/article/10.3390/plants11141826/s1, Supplementary: Table S1: ANOVA of root-related traits of B. napus for the three combined replications (R1, R2, and R3); Table S2: Summary of significant SNPs associated with root-related traits in *B. napus* detected by six models under low-potassium conditions; Table S3: Summary of QTN clusters associated with root-related traits in *B. napus* detected under low-potassium conditions; Table S4: List of pleiotropic QTN clusters detected in 327 *B. napus* accessions; Table S5: List of all potential candidate genes within 100kb upstream and downstream of the lead SNPs; Table S6: GO enrichment results of the candidate genes; Table S7: KEGG enrichment results of the candidate genes; Table S8: Information on lead SNPs overlapped with SNPs reported by Li et al. (2021). Supplementary: Figure S1; (A) Shoot fresh weight of the 8 lines across the 9 different K concentrations (B) The ratio of SFW between the stress treatment and the control condition; Figure S2: Principle component analysis of the nine studied traits: (A) displays the loading plots of the two principal components, (B) the Eigenvalues of the principal component, (C) depicts the individual and cumulative contribution of each principal component; Figure S3: Quantile–quantile plots of estimated −log_10_ (*p*) from phenotype–genotype association analysis of twelve root-related traits using six multilocus GWAS methods; Figure S4: Functional annotation of the detected candidate’s genes. (A) GO classification of the genes, (B) GO analysis of the top 30 genes, (C) KEGG analysis of the top 30 genes; Figure S5: Network of protein interactions. The gene connections were suggested by the network. Proteins are represented by network nodes, while query proteins and the initial shell of an interactor are represented by colored nodes.

## Abbreviations

GWAS: genome-wide association study; ML, multilocus; EMMA, efficient mixed-model association; QTN, quantitative-trait nucleotide; QTL, quantitative-trait loci; MLM, mixed linear model; GLM, marker–trait associations; MTAs, SNP, single-nucleotide polymorphism; logarithm of odds; LOD, LD, linkage disequilibrium; RSA, root-system architecture; qRT, cluster of root trait; PVE, phenotypic variance explained; Q-Q plot, quantile–quantile plot; MAF, minor allele frequency; GO, gene ontology; KEGG, Kyoto Encyclopedia of Genes and Genomes; KUE, potassium utilization efficiency; RM, root morphology; LK, low potassium; qc, QTL cluster.
