# Supplementary material for: Genome-Wide Association Studies of Root-Related Traits in Brassica napus L. under Low-Potassium Conditions
Source: Plants (Basel). 2022 Jul 12;11(14):1826. doi: 10.3390/plants11141826 (PMC9318150; doi:10.3390/plants11141826)
Supplement: Supplementary file 1 [file plants-11-01826-s001.zip › plants-1769147-supplementary.pdf]

Figure S1

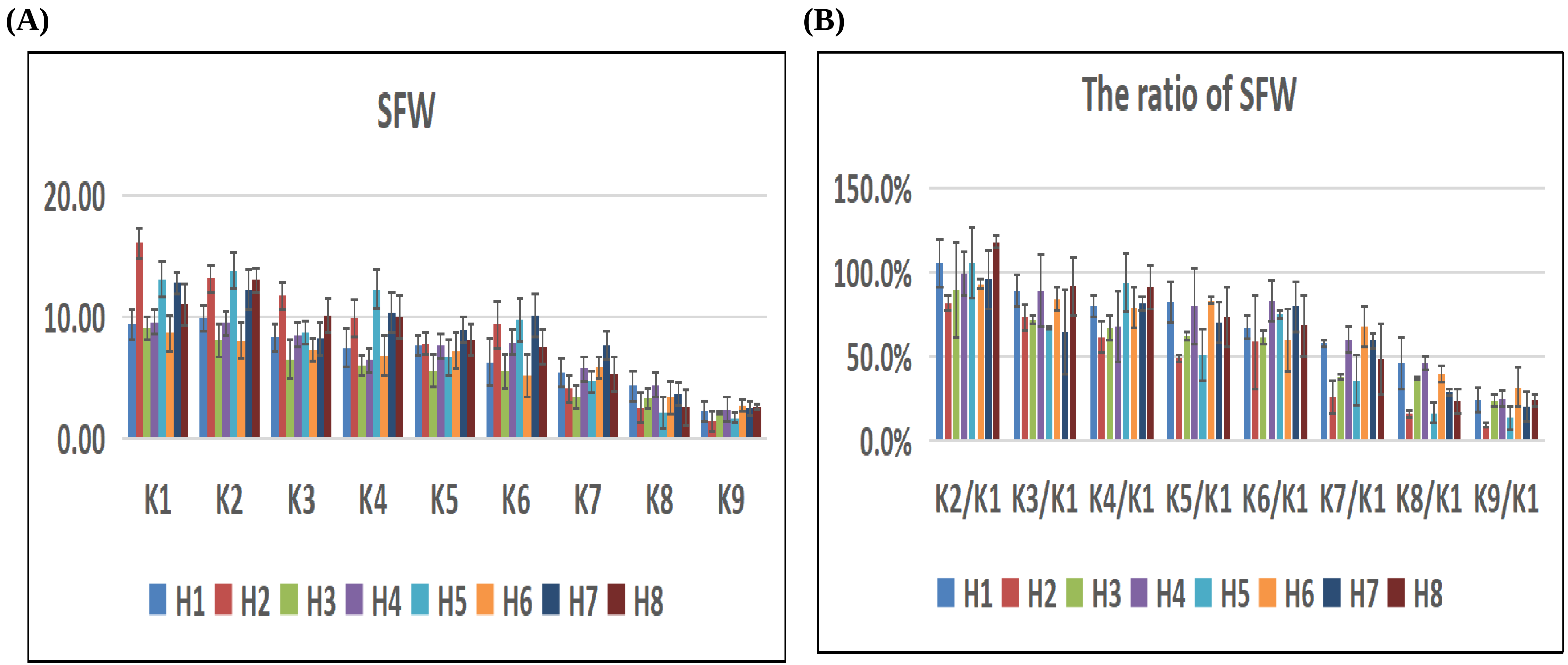

(A) Shoot fresh weight of the 8 lines across the 9 different K concentrations (B) The ratio of SFW between the stress treatment and the control condition

Figure S2

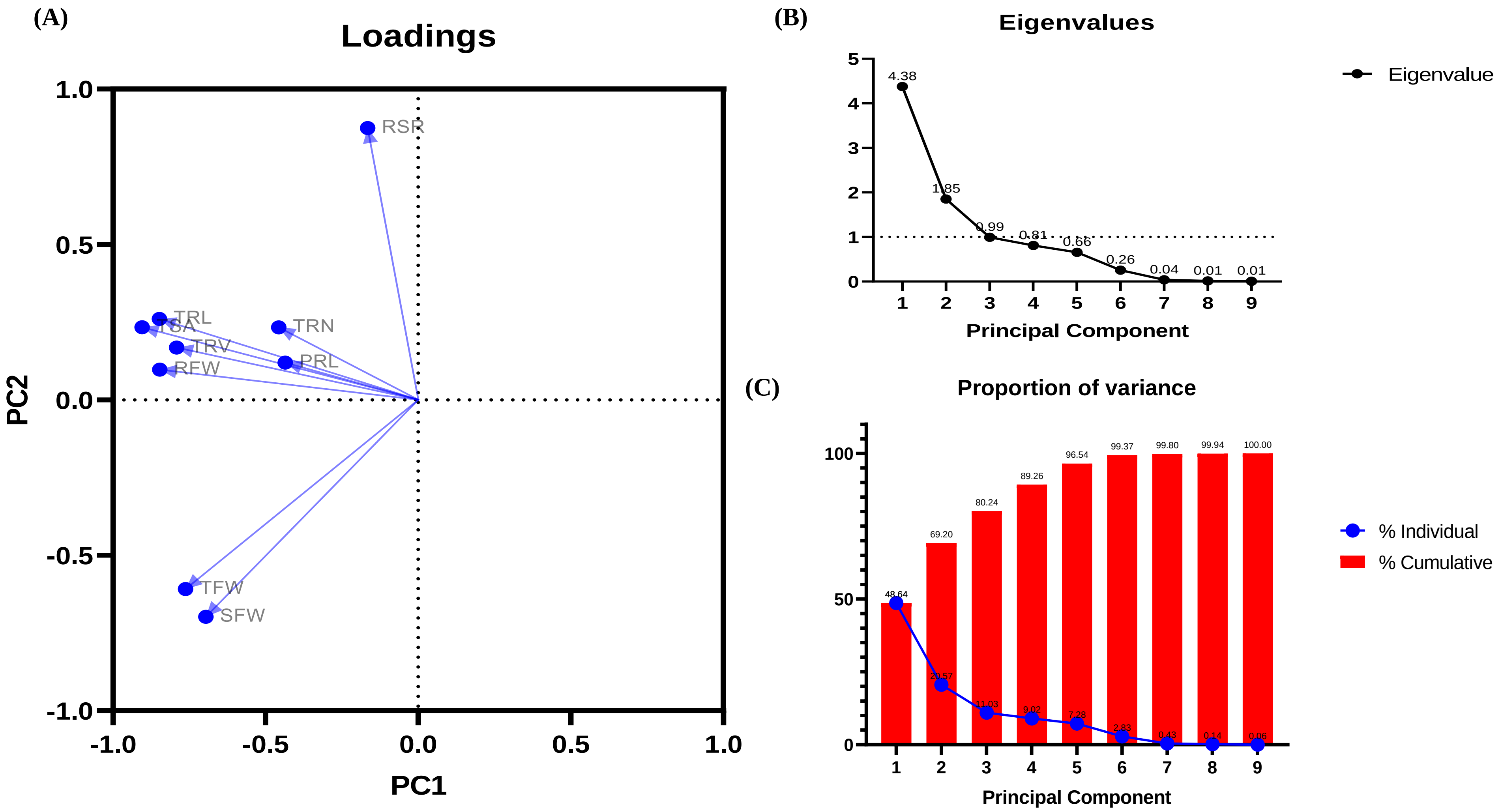

Principle Component Analysis of the nine studied traits: (A) Displays the loading plots of the two principal components (B) the Eigenvalues of the principal component(C) Depicts the individual and cumulative contribution of each principal component

Figure S3

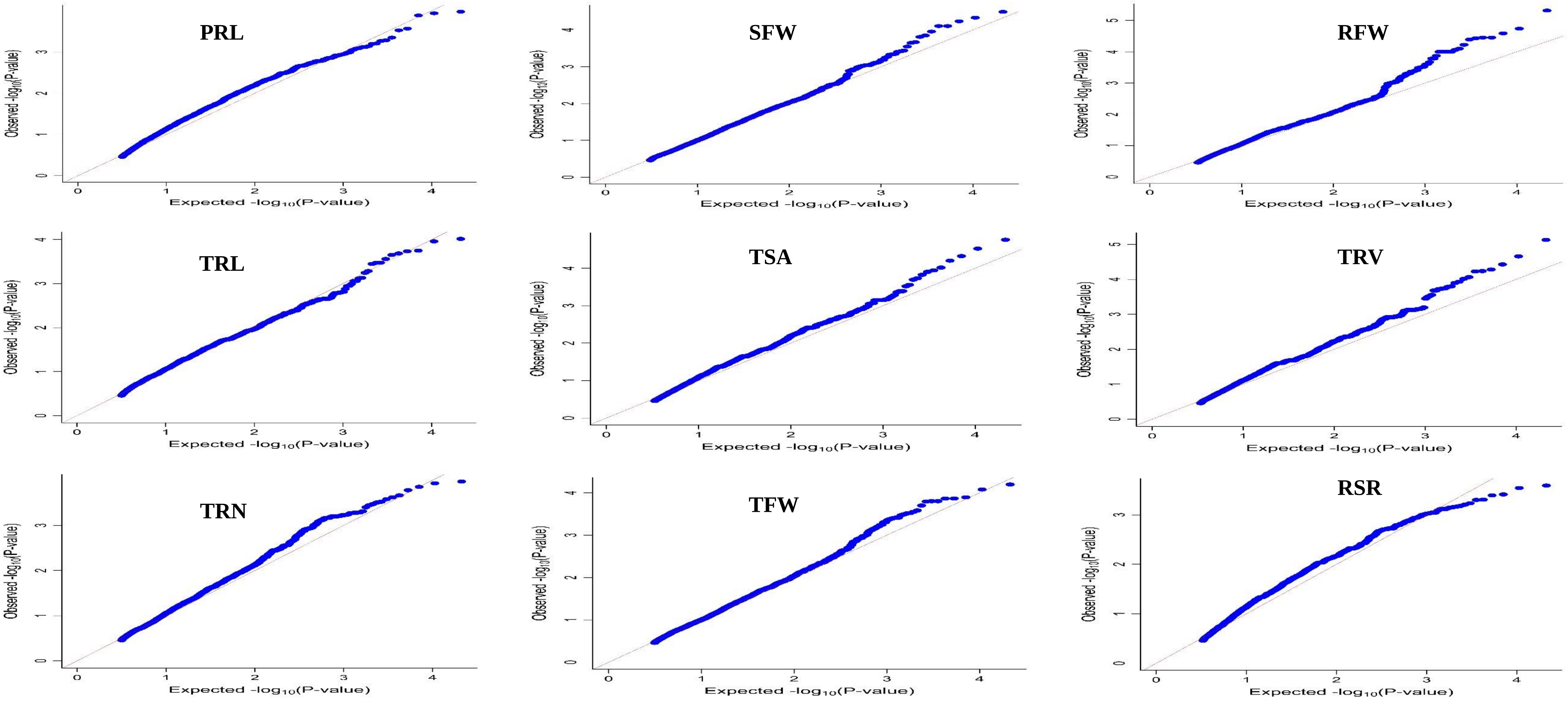

Quantile-quantile plots of estimated $-\log_{10}$ *(P)* from Phenotype-genotype association analysis of twelve root-related traits using six multi-locus GWAS methods.

Figure S4

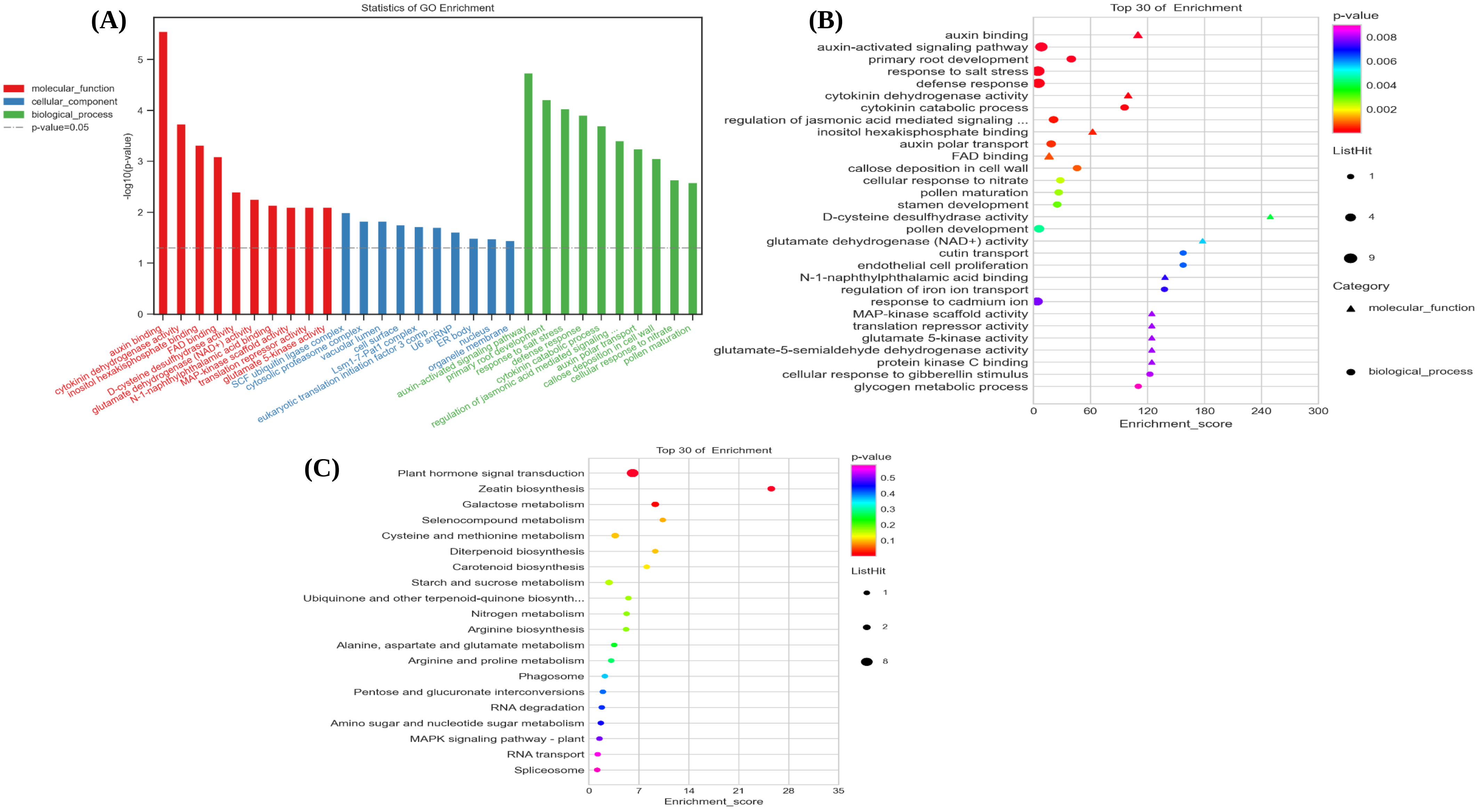

Functional annotation of the detected candidates genes. (A) GO classification of the genes (B) GO analysis of the top 30 genes (C) KEGG analysis of the top 30 gene

Figure S5

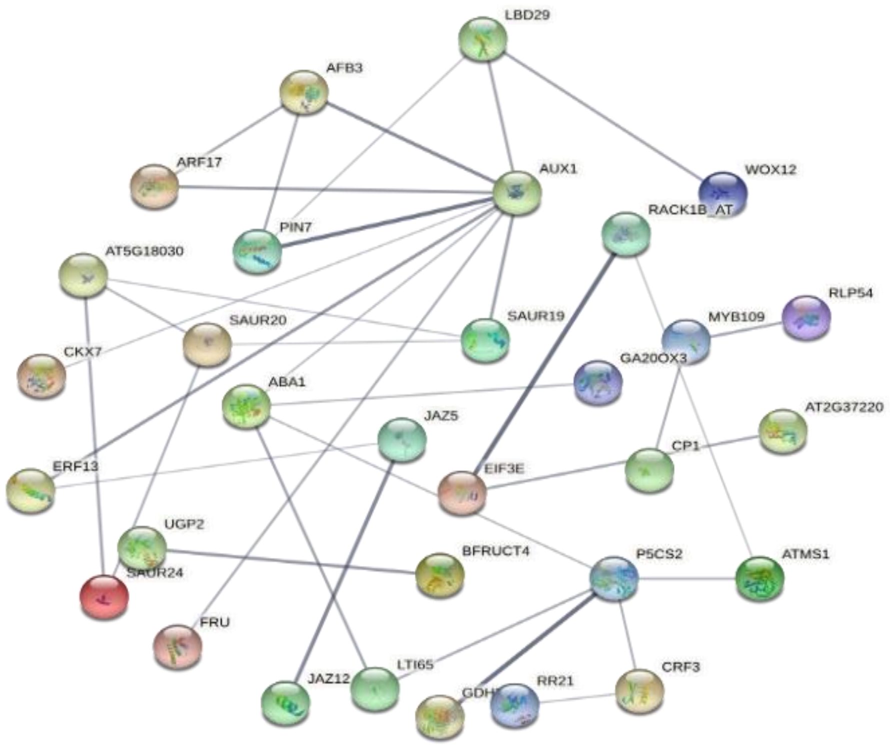

Network of protein interactions. The gene connections were suggested by the network. Proteins are represented by network nodes, while query proteins and the initial shell of an interactor are represented by colored nodes
